# Multi-layer process control in selective laser melting: a reinforcement learning approach

**DOI:** 10.1007/s10845-024-02548-3

**Published:** 2024-12-18

**Authors:** Stylianos Vagenas, Taha Al-Saadi, George Panoutsos

**Affiliations:** Department of Automatic Control & Systems Engineering, University of Sheffield, Sheffield, UK

**Keywords:** Powder bed fusion, Selective laser melting, Ti–6Al–4V, Process control, Reinforcement learning

## Abstract

Powder bed fusion (PBF) is an original additive manufacturing technique for creating 3D parts layer-by-layer. While there are numerous benefits to this process, the complex undergoing physical phenomena are challenging to analytically model and interpret. Hence, integrated and control-oriented 3D models are lacking in the current literature. As a result, the state of the art in process control for the powder bed fusion (PBF) process is not as advanced as in other manufacturing processes. Reinforcement learning is a machine learning, data-driven mathematical and computational framework that can be used for process control while addressing this challenge (lack of control-oriented models) effectively. Its flexible formulation and its trial-and-error nature make reinforcement learning suitable for processes where the model is intricate or even unknown. The focus of this research work is selective laser melting, which is a laser-based PBF process. For the first time in the literature we demonstrate the benefits of a reinforcement learning process control framework for multiple layers (complete 3D parts) and we highlight the importance of stability during training. The presented case studies confirm the effectiveness of the proposed control framework, directly addressing heat accumulation issues while demonstrating effective overall process control, hence opening up opportunities for further research and impact in this area.

## Introduction

Powder bed fusion (PBF) stands out as an innovative approach to metal additive manufacturing (AM), attracting considerable attention from both academia and industry. This manufacturing method, based on depositing layers of material using 3D computer designs, offers notable advantages compared to traditional manufacturing techniques, see Dev Singh et al. (2021). PBF allows for the creation of intricate metal components, see Liu and Shin (2019), with complex shapes and microstructures. In contrast to traditionally manufactured metal parts that typically require multiple processes such as drilling and welding, PBF achieves similar results in a single process, leading to a decreased reliance on various tools, see Huang et al. (2016).

PBF consists of two main manufacturing techniques referred to as selective laser melting (SLM) and electron beam melting (EBM), see Dev Singh et al. (2021). In both SLM and EBM, an energy source, whether it be a laser or an electron beam, see Liu and Shin (2019), selectively melts the powder distributed on the build platform. This study focuses on the SLM process, as it is presently the most widely used and commercialised. As discussed in the following sections, existing SLM modelling efforts frequently offer a complex physics or data-driven representation of the real process. These versions, while realistic, are not suitable for applying advanced control due to their complexity and/or computational cost. Hence, there is a research gap in control-oriented, integrated, and computationally fast models, capable of establishing the relationship between desired process characteristics and controllable parameters across all relevant scales. Consequently, in industry, manufacturers resort to optimisation based on experience, without incorporating any active feedback process control, or merely incorporating simple feedforward and predetermined fixed control profiles that do not fully exploit the advantages of the process.

Simple part geometries, combined with simple control targets (e.g. constant temperature) could be addressed sufficiently with simple control methods. However, as part geometry becomes more complex, process and part models become more realistic and the control targets become more intricate, sophisticated control methods are needed that often require models and strict assumptions (e.g. on sensing methods). In SLM, such models (e.g. integrated, across scales) do not exist, or sensing and signal property assumptions cannot be satisfied. Using machine learning to control the process is an alternative, for example via the use of reinforcement learning (RL). RL offers a straightforward control framework, often implemented as iterative optimisation, for which model development and signal property assumptions can be less strict. For example, Nian et al. (2020) discuss the potential of RL for industrial process control, and Ogoke and Farimani (2021) demonstrate a RL framework for printing a single layer for a SLM process. In this study, for the first time in the literature, the control of a SLM process across multiple layers (complete 3D parts) is investigated via RL. The resulting framework is benchmarked against Proportional-Integral-Derivative (PID) control. This work reflects on the advantages as well as the limitations of RL and the necessity for stability in SLM process control, see Vagenas and Panoutsos (2023), hence, the investigation is extended to include a new, stable RL variant.

The purpose of this research study is to develop and evaluate a process control framework based on a control-oriented 3D model of the SLM process. The 3D model represents the multi-layer SLM process, with particular emphasis on demonstrating - as an example - the challenges associated with heat accumulation among the layers as the number of layers increases. It is shown that the absence of a controller leads to heat accumulation issues, hence feedback control is considered. As a result of this investigation, a RL framework is proposed as a control method to adjust the power of the heat source based on process monitoring and feedback. The proposed methods are benchmarked against a carefully tuned PID controller. The main contributions and remarks of this work are the following:

- Further development of an existing 2D SLM model to a new 3D one, and implementation of process control on a simulated 3D SLM platform.
- Demonstration of the benefits and limitations of a layer-wise control approach in 3D SLM, for simple and more complex control objectives (target tracking).
- Reveal and reflect upon the benefits and limitations of a RL control approach, compared to traditional control theory-based methods.
- A new, stable RL framework is proposed and a demonstration of its benefits in the stability of the RL training and in the RL performance is included.

## SLM modelling

### The SLM process

**Fig. 1:**
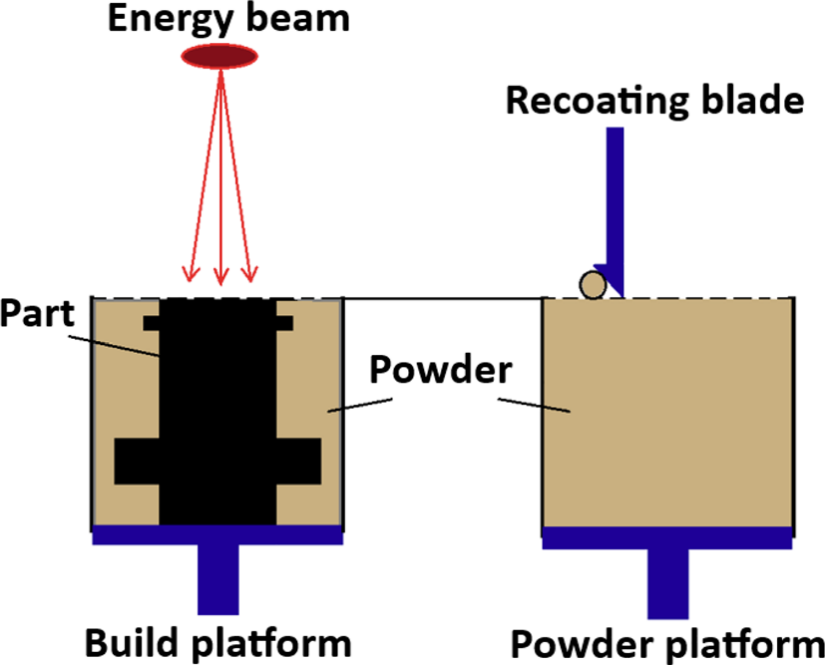
Schematic of a SLM machine set up

SLM manufacturing machines, also known as laser PBF machines, utilise metallic materials (e.g. titanium alloys) in powder form. This metal powder is stored on the powder platform and it is swept onto the build platform by a recoating blade. Then, the laser beam heat source selectively melts the powder on the build platform following a specified scanning pattern. Afterwards, the build platform is lowered and the recoating blade sweeps a second layer of powder onto the build platform. This process is repeated until the final part is completed, see Moylan et al. (2014). Figure 1 presents a schematic of the SLM set up, including the aforementioned features.

Extensive research has been dedicated to understanding the physics behind SLM processes, with a particular emphasis on the meltpool temperature, see Shi et al. (2019) and Wang et al. (2020), as this factor strongly correlates with the SLM part’s quality. The meltpool refers to the region at the interface between the laser and the metal powder, where the powder particles fuse together to create a pool of molten metal. Once the laser beam moves to a different location, the meltpool solidifies, see Liu and Shin (2019). The meltpool’s characteristics have great impact on the density and the microstructure of the component. Maintaining a uniform and consistent meltpool temperature and shape throughout the SLM build is crucial for preventing major defects, such as the formation of keyhole effects, see Snell et al. (2019). Heat accumulation among the layers of the SLM build would violate this goal of uniform and consistent meltpool temperatures and shapes. Hence, in this work, the heat accumulation challenge is selected as the primary objective to address.

### SLM modelling efforts

During the manufacturing process, the metallic powder undergoes multiple transitions, from a powder form to a liquid state and then rapidly solidifying into a dense metal. These are very complex behaviours that make it challenging to create an integrated and precise model that accurately describes the relationship between the process inputs and the final part’s characteristics analytically. However, there have been significant efforts in the literature to develop models specifically focusing on the SLM manufacturing process. These modelling attempts involve combining analytical modelling efforts with numerical techniques such as the Finite Element Method (FEM), as well as data-driven modelling methods.

In a study conducted by Matsumoto et al. (2002), a technique was introduced to calculate the temperature distribution and stress within a single-layer build during the SLM process. The approach involved analytical physics formulations, and 2D FEM was employed for the necessary calculations. Roberts et al. (2009) employed an innovative simulation technique using 3D FEM modelling in their research. The simulation results indicated that “the heating and subsequent cooling to the ambient temperature occur within a few tenths of a millisecond of each other, thus suggesting that the irradiated spots are subject to rapid thermal cycles. These rapid cycles are associated with commensurate thermal stress changes” (Roberts et al., 2009). Megahed et al. (2016) analysed the modelling framework of PBF processes, with a specific focus on SLM. The authors introduced analytical equations for physics-based modelling and utilised FEM to solve these models. Shi et al. (2019) developed a comprehensive multi-physics and multi-scale framework that incorporated 3D modelling of heat transfer, flow dynamics, and considerations of grain size and microstructure. Numerical methods, including FEM, were employed to solve the equations of mass, momentum, and energy conservation. Wang et al. (2020) created a control-oriented model to analyse the temperature and the dynamics of the cross-sectional area of the meltpool when scanning a part with multiple tracks (single-layer builds). Subsequently, a controller was designed with the objective of modifying the laser power. In the following sections, the work of Wang et al. (2020) is used as a starting point and the 2D model is extended to a 3D one, in an attempt to establish a fully controllable SLM multi-layer model.

Regarding data-driven modelling approaches, Tapia et al. (2018) suggested the creation of a material database that could describe robust concepts within the SLM process. The developed model was capable of predicting the meltpool depth based on input parameters such as the power of the energy beam. It was found to perform sufficiently even when the training data was sub-optimal, as long as appropriate physics filters were employed. Kouraytem et al. (2021) presented a compilation of analytical and data-driven modelling methods, highlighting the increasing prevalence of data-driven models. The study emphasised the significance of machine learning as a data processing technique to support data-driven modelling efforts. Finally, a more comprehensive review on modelling attempts can be found in the the work of Soundararajan et al. (2021).

### Extending a 2D SLM model to 3D

**Fig. 2:**
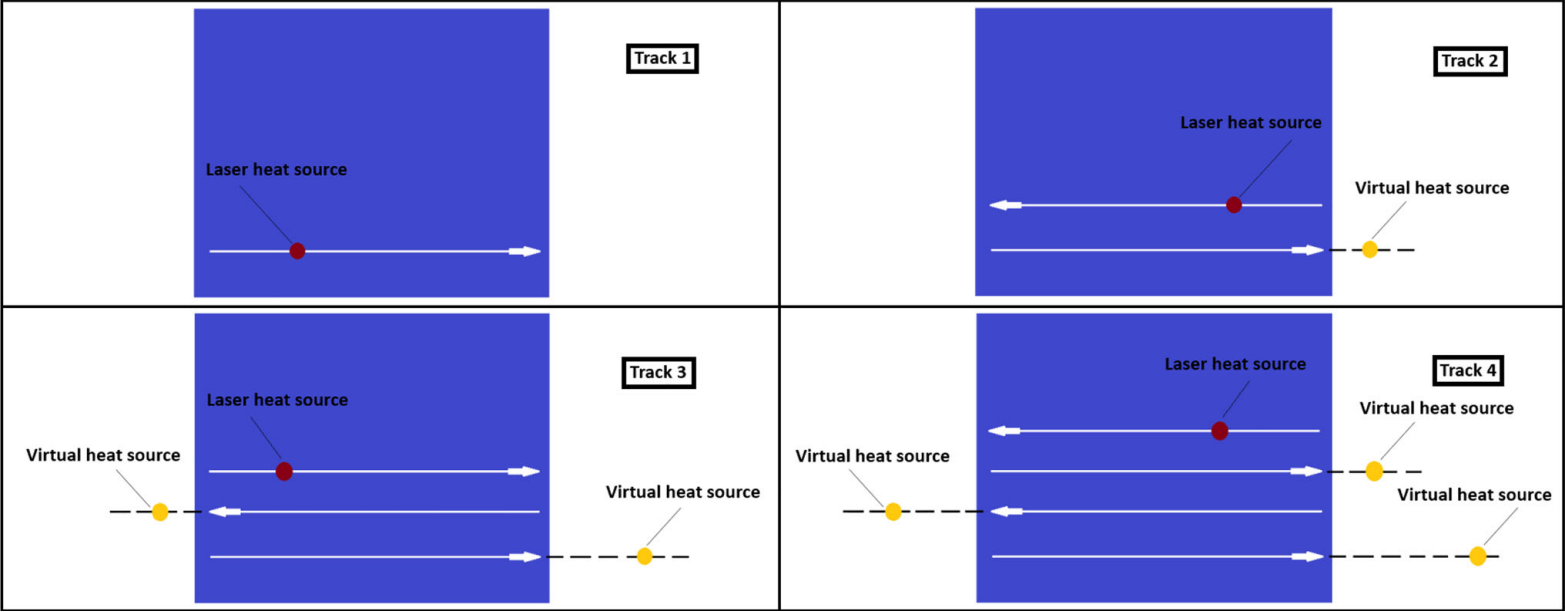
Visualisation of the build of a layer, track per track. Example for a layer of 4 tracks

For the model used in this study, the work of Wang et al. (2020) is used as a template; the authors successfully created a control-oriented 2D model of a SLM process. The developed 2D model is based on building multiple tracks on a layer, following a back and forth scanning pattern. This model is extended, so that each time a new track is built, the model takes into account the heat accumulation due to the previous track, utilising the concept of a virtual heat source and applying the Rosenthal solution, as introduced in Rosenthal (1941). This virtual source concept is shown in Fig. 2. The material used in this work is Ti–6Al–4V powder and the manufacturing properties used are presented in Table 1.

**Fig. 3:**
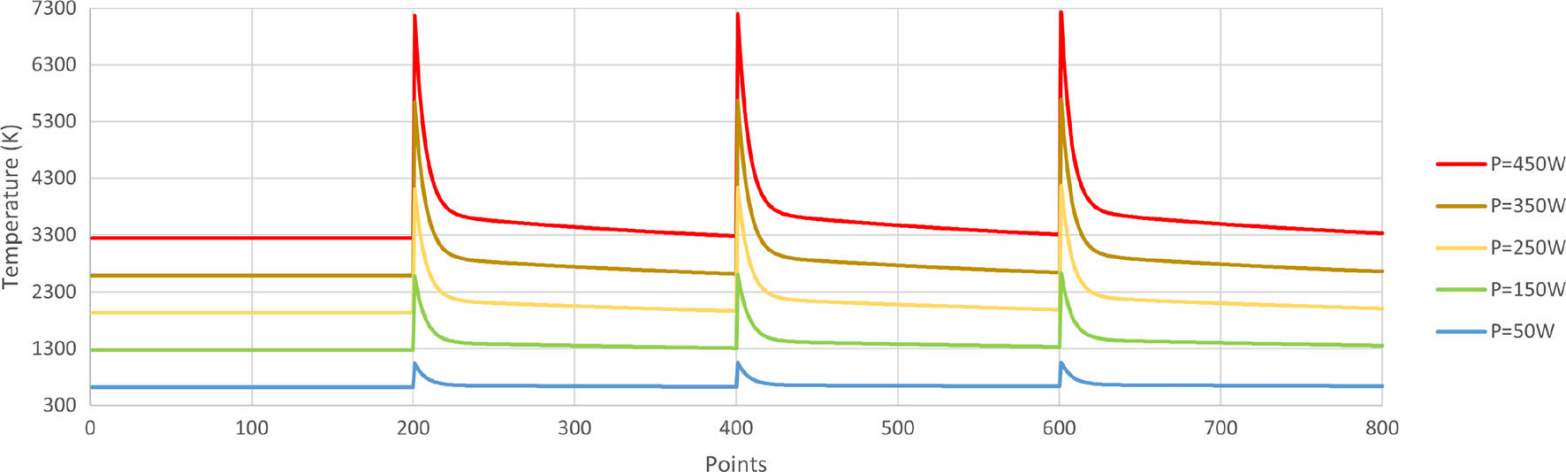
Temperature history collected for different values of power, *P*, during a single-layer, 4-track build (10 mm length for each track)

In order to investigate the temperature behaviour within a layer, an example for a 4-track build is investigated, with 10 mm length for each track, on a single layer. The layer consists of 800 points, which show the meltpool temperature history. The temperature observed is shown in Fig. 3. As it is observed, the first track is built at a constant temperature, as there is no heat accumulation effect yet. From the second track onwards, temperature peaks are observed at the beginning of each track, due to the scanning strategy (beam passing next to recently scanned material) and the resulting overall heat effect of the previous tracks. Moreover, these temperature peaks tend to reach higher values as the building of the part progresses to further tracks built on the same layer. Given enough time, and a long enough track, the temperature within a track starts saturating towards the value that corresponds to the temperature that would have been achieved without the heat effect of the previous tracks.

**Table 1:** SLM model parameters

Parameter	Symbol	Value
Melting temperature (K)	$T_{m}$	1923
Ambient temperature (K)	$T_{a}$	292
Layer thickness ($\upmu$m)	$L_{th}$	30
Scanning speed (mm/s)	*V*	800
Sampling rate ($\upmu$s/point)	$t_{s}$	62.5

Regarding heat accumulation, the Rosenthal solution is used, as introduced in Rosenthal (1941), in order to estimate the heat effect of an already built layer to the next layer being built. In addition, a delay of five seconds is assumed between the end of one layer and the beginning of the next one, due to the time required for spreading the new powder after the completion of each layer. As a result, a 3D SLM model is produced, which does not only demonstrate how heat accumulates within each layer (2D), but among all the layers in height as well (3D).

**Fig. 4:**
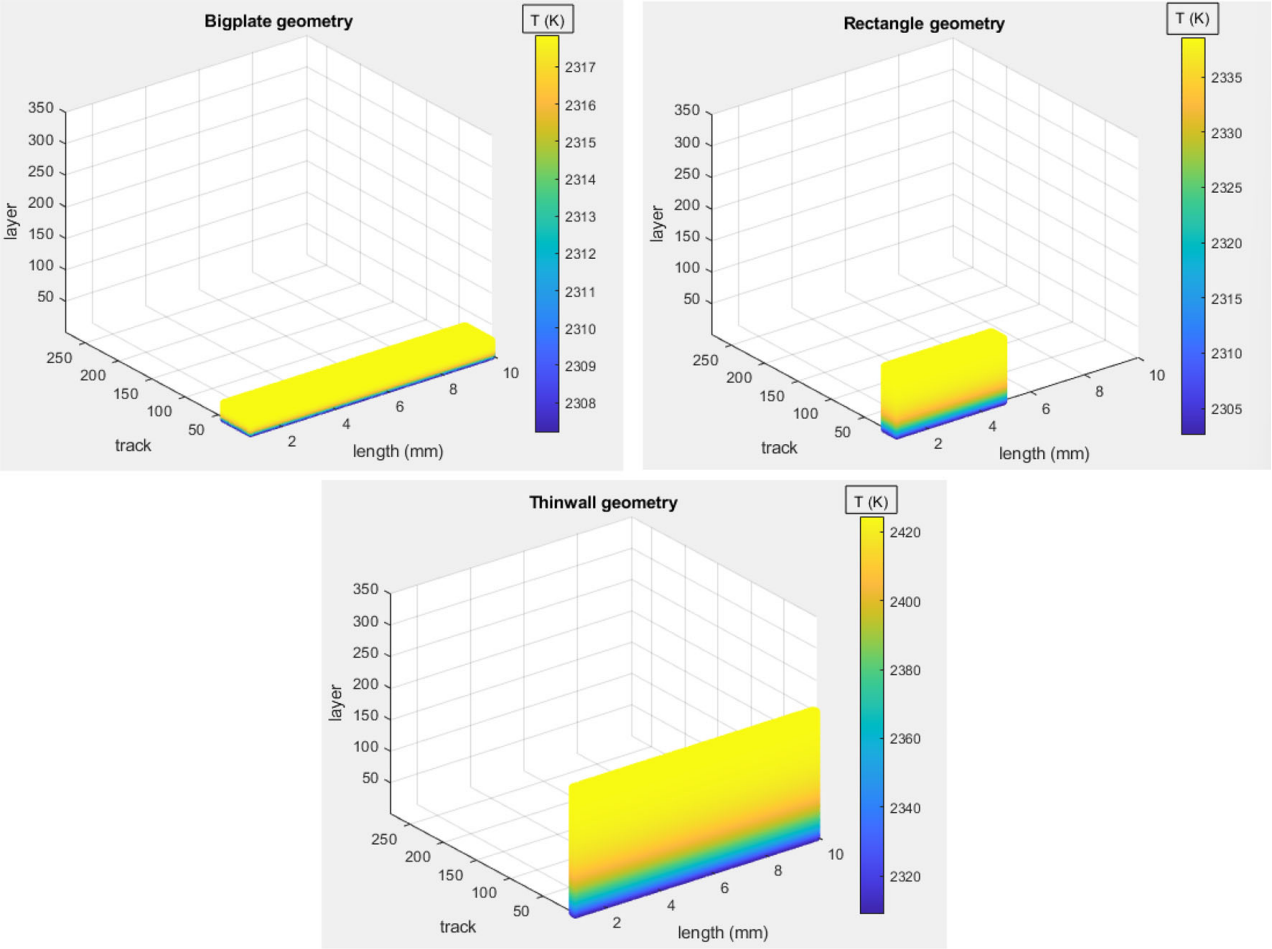
Colormaps for visual representation of heat accumulation among the layers. Each point is denoted with the average temperature of the layer in which it belongs. Axes are in scale so that visual dimension differences correspond to actual dimension differences among the three geometries (Color figure online)

**Fig. 5:**
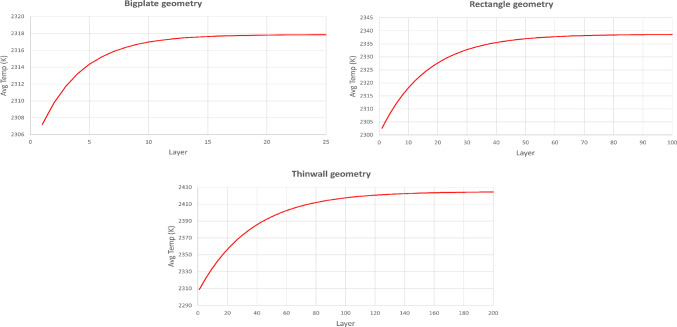
Average layer temperature graphs for representation of heat accumulation among the layers. These graphs correspond to the respective colormaps in Fig. 4

In order to gain some practical intuition about the 3D SLM model’s temperature behaviour, simulations corresponding to three different geometries are produced. The geometries are distinguished by the dimensions of the layer of the build, as *bigplate* (40 tracks, 10 mm each), *rectangle* (18 tracks, 5 mm each), and *thinwall* (4 tracks, 10 mm each) geometry. The melting temperature of the material is 1923 K. However, in the SLM model developed by Wang et al. (2020), it is assumed that the steady-state temperature of the meltpool is a constant percentage higher than the melting temperature. As implemented by Wang et al. (2020), the value of 20% above the melting point of the material is chosen in this study, which is 2308 K. For each geometry, the laser power, *P*, is set to an integer value that approximately corresponds to an average layer temperature of 2308 K. For the *bigplate*, the power is set as *P* = 258 W, for the *rectangle*, the power is set as *P* = 198 K, and for the *thinwall*, the power is set as *P* = 280 W. As seen in Figs. 4 and 5 the average layer temperature of the first layer in all cases is approximately 2308 K. As the build progresses and more layers are added, the average layer temperature increases because of heat accumulation. In all three scenarios, as many layers as necessary are simulated for the heat accumulation among the layers to reach a saturation point, so that one can appreciate the significance of the geometry of the build in heat accumulation issues.

It is observed, that the *bigplate* geometry’s heat accumulation saturates after 25 layers, the *rectangle* geometry’s after 100 layers, and the *thinwall* geometry’s after 200 layers. Moreover, it is measured that the average layer temperature difference between starting layer and saturation layer in the *bigplate* geometry is 10 K, while in the *rectangle* geometry is 36 K and in the *thinwall* geometry is 115 K. Hence, it can be concluded that the *thinwall* geometry comprises the most challenging geometry regarding layer-wise heat accumulation issues. Therefore, the *thinwall* geometry is selected for the following process control case studies, in an attempt to clearly demonstrate the adjustment of the power accordingly on each layer, with a layer-wise control approach.

## Process control

### The need for feedback control in PBF

Process control is a critical task in relevant industry sectors, see Juneja et al. (2021). Within the PBF context it requires special attention to enable the process to realise its potential in terms of achieving bespoke microstructures, ensuring stability (e.g. for certification of aerospace parts), see Vagenas and Panoutsos (2023) and Jensen et al. (2023), optimising surface and mechanical characteristics in complex designs etc. In this work, the focus is on process control techniques that are based on control theory, as well as on data-driven approaches in order to determine the benefits and the drawbacks for each class of methods. Control theory methods such as PID and feedforward control are the most popular approaches in industrial process control, mainly due to the simplicity of implementation, see Nian et al. (2020). The fundamental principle of the PID control, see Åström and Hägglund (2006), involves measuring the difference between the actual and desired system output signals. The PID controller then takes an action in order to minimise this difference, based on the dynamic characteristics of the error signal ($K_P$, $K_I$ and $K_D$ terms in the control law). These are well-established, interpretable methods that can be applied in a large variety of tasks. When addressing a simplified model of PBF, where the complexity is low and the control target is simple, these theory methods seem to be the obvious choice as a first attempt to apply process feedback control in PBF. However, when the part geometry and underlying models become more complex and the control target is not as straightforward (e.g. multiple target tracking), simple control theory methods would not perform well in PBF (e.g. to counteract disturbances, signal delays, process drifts etc.). In this case, the most valuable information is monitoring and process data, in order to identify the relationship between the control system’s inputs and outputs. Hence, data-driven approaches and feedback control methods could help alleviate some of the aforementioned challenges.

### Process control in PBF

In this section, attempts at process feedback control for PBF in the literature are appraised. Mireles et al. (2015) presented a feedback control method based on a LabVIEW virtual instrument for the EBM process. They altered the process temperature, in order to achieve a desired grain size. The authors used a virtual instrument to apply control with feedback obtained via IR camera images and layer information decoded from calculations made by the virtual instrument’s loop iterations. However, no systematic feedback control law was presented. Renken et al. (2018) presented an approach of a P-controller which enabled fast control of the meltpool temperature. This approach reduced the deviation of process temperature by up to 73%, which led to more stable conditions in the meltpool, in comparison with constant laser power strategies. Based on this approach, Renken et al. (2019) presented an improved method using a model-assisted version of the earlier attempt that reduced the deviation of process temperature by up to 90%. Kruth et al. (2007) utilised a PID control system for overhang structures, with a high-speed camera installed on the SLM machine. The control system was represented as a single-input-single-output system. Wang et al. (2020) introduced a feedforward controller to address overheating issues and keyhole effects in SLM. The purpose of their controller was to maintain the meltpool cross-sectional area at a constant level throughout the building process. Liao-McPherson et al. (2022) introduced a control-oriented thermal model of a multi-layer SLM process and proposed an in-layer Linear-Quadratic Regulator (LQR) to track temperature. Vasileska et al. (2020) were one of the first teams to attempt a layer-wise control strategy in SLM. They could effectively apply control to limit geometrical defects due to overheating. Kavas et al. (2023) developed a robust controller, inspired by iterative learning control and online feedback optimisation, which altered the laser power in order to stabilise the inter-layer temperature. In general, the recent work of Lupi et al. (2023) covers control requirements in AM, for the reader to gain a broader perspective.

The aforementioned control techniques are either too simple to be effective at scale (e.g. PID) when part and process complexity increases, or require tuning which is not trivial (e.g. LQR) and strong assumptions (e.g. regarding disturbances, process drift), as discussed in the introduction section of this study. On the other hand, RL offers a data-driven control framework, for which learning algorithms are used to create an effective control policy. There seems to be still little evidence of RL-based process control in PBF processes. State of the art RL attempts are still in a preliminary development phase, focusing mostly on single-layer parts or applied in other AM processes (e.g. blown powder), but not yet in PBF. In PBF, Ogoke and Farimani (2021), influenced by the contributions of Eagar and Tsai (1983) in process modelling, and Wolfer et al. (2019), created a simulation model that can track the temperature history that is created by a travelling laser beam, on a single-layer part, and implemented RL control to achieve a desired meltpool depth. Building on this work, Vagenas and Panoutsos (2023) replicated the above results and demonstrated the need for stability in the behaviour of RL process control in PBF.

The above literature review shows that there are mostly simple feedback and PID-based approaches for PBF process feedback control, while data-driven approaches based on RL have been only recently investigated in a preliminary fashion. Moreover, there is no evidence in comparing control theory with data-driven techniques, particularly in multi-layer PBF environments. Hence, benchmarking of these methods is needed, and a comparison between control theory and data-driven techniques is to provide substantial intuition on multi-layer PBF control.

### Reinforcement learning overview

RL is a data-driven iterative optimisation approach centred around interactions with an environment/process. The controller, also known as the agent, serves as a decision maker, consistently learning and refining its control actions. Unlike being explicitly instructed which actions to take in specific states, the RL agent must discover the most effective actions through a process of trial-and-error, see Sutton and Barto (1998). This exploration is guided by the objective of maximising a numerical target, the reward.

**Fig. 6:**
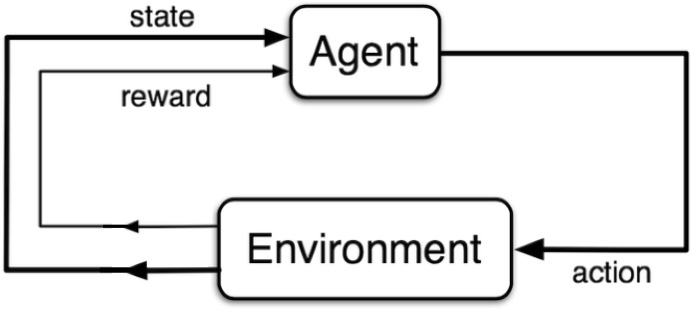
The RL paradigm

A high-level diagrammatic representation of RL is shown in Fig. 6. The RL sequence starts with the agent being in an initial set of states, $s_t$, with *t* denoting the corresponding timestep. The agent takes an action, $a_t$, in the environment’s initial states, and the response of the environment is fed back to the agent with the form of a new set of states, $s_{t+1}$, and a corresponding reward, $r_{t+1}$. For example, within the context of PBF, the action could be the laser power of a SLM process, while the reward could cover maintaining a constant layer temperature.

**Fig. 7:**
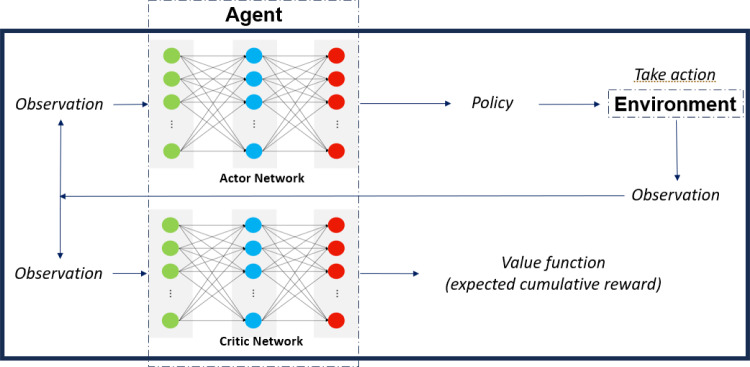
The actor critic framework

The most popular and efficient approach, in terms of process control in RL, is the actor critic class of methods, see Schulman et al. (2017); Haarnoja et al. (2018). In Fig. 7, a generic structure is shown for the control framework. In the actor critic class of RL, the agent can be formulated as a pair of neural networks, each of which has a very specific role to play. The neural networks are function approximators (policy function and value function respectively) and are denoted with $\pi _\theta (s,a)$ for the actor network, parameterised by $\theta$, that approximates the policy distribution, and $v_w(s)$ for the critic network, parameterised by *w*, that approximates the value. The magnitude of the update step that the neural networks go through during the learning process is dependent on the learning rate, denoted by $\alpha$ for the actor network, and $\beta$ for the critic network.

### Soft actor critic method

The soft actor critic (SAC) algorithm, introduced by Haarnoja et al. (2018), is a model-free actor critic method within the RL domain. It aims not only to maximise expected rewards but also to maximise entropy (via entropy regularisation), which enhances the exploration of control actions. Considered as a state of the art RL algorithm, SAC has demonstrated exceptional performance in continuous control benchmarks, see Pardo (2020). It employs an experience replay buffer to store previously collected agent-environment interactions, utilising them to enhance sample efficiency. The SAC algorithm consists of an actor neural network responsible for policy updates, determining action selection, and two critic neural networks responsible for evaluating the quality of the taken action. It balances exploration and exploitation, using an entropy coefficient (or reward scale) and this hyperparameter is found to be the only hyperparameter that needs to be tuned for SAC to perform. For this work, the SAC algorithm is used, as in the *stable-baselines3* platform by Raffin et al. (2021).

### Proposed method: adaptive weighted actor critic

Despite the benefits of the actor critic framework, stability remains a key challenge in RL process control, see Nian et al. (2020) and Vagenas and Panoutsos (2023), as there are no performance guarantees. In an attempt to introduce more stable RL frameworks for PBF, for the first time the Adaptive Weighted Actor Critic (AWAC) algorithm is introduced. AWAC is a model-free actor critic method which aims to improve the stability of the agent’s training. It utilises an auto-tuned signal from the environment, which guides the agent towards its goal while stability is accounted for.

In the *advantage actor critic* learning process, see Mnih et al. (2016), a metric called *advantage* is defined, this is *A*(*s*, *a*), as presented in (1). The actor network’s update rule is presented in (2), while the critic network’s update rule is presented in (3). The factor $\gamma$ is a numerical value, such as $0\le \gamma \le 1$, and determines the current contribution of future returns. The value of this discount factor is usually set empirically as $\gamma =0.99$.

1 $$\begin{aligned} A(s_t,a_t)= &  r_{t+1} + \gamma v_w(s_{t+1})-v_w(s_t) \end{aligned}$$

2 $$\begin{aligned} \Delta \theta= &  \alpha \nabla _\theta (ln\pi _\theta (s_t,a_t))A(s_t,a_t) \end{aligned}$$

3 $$\begin{aligned} \Delta w= &  \beta \nabla _w(v_w(s_t))A(s_t,a_t) \end{aligned}$$

At the start of the process, the agent has an initial set of states, $s_{t}$. The critic network approximates the corresponding value, $v_w(s_t)$. The agent takes an action $a_{t}$ in the environment, sampled from the actor network $\pi _\theta (s_t,a_t)$. A new set of states, $s_{t+1}$, with the corresponding reward, $r_{t+1}$, are fed back to it and the critic network, yet again, approximates the corresponding value $v_w(s_{t+1})$. Having this information, one can now calculate $A(s_t,a_t)$ and the update steps $\Delta \theta$ and $\Delta w$ of the neural networks. The aim of this learning process is for the agent to be able to choose actions that lead to higher returns.

The main idea behind AWAC is the construction of a cost function, $c_{t+1}$, in which stability is accounted for, just as the reward function relates to performance. This cost function is a positive defined function and its formulation depends on the task at hand. This cost function is used to design a discount for the calculated advantage, $A(s_t,a_t)$, so that the agent is guided to perform in a stable manner. The novelty of the proposed approach is within the way this discount is designed.

**Fig. 8:**
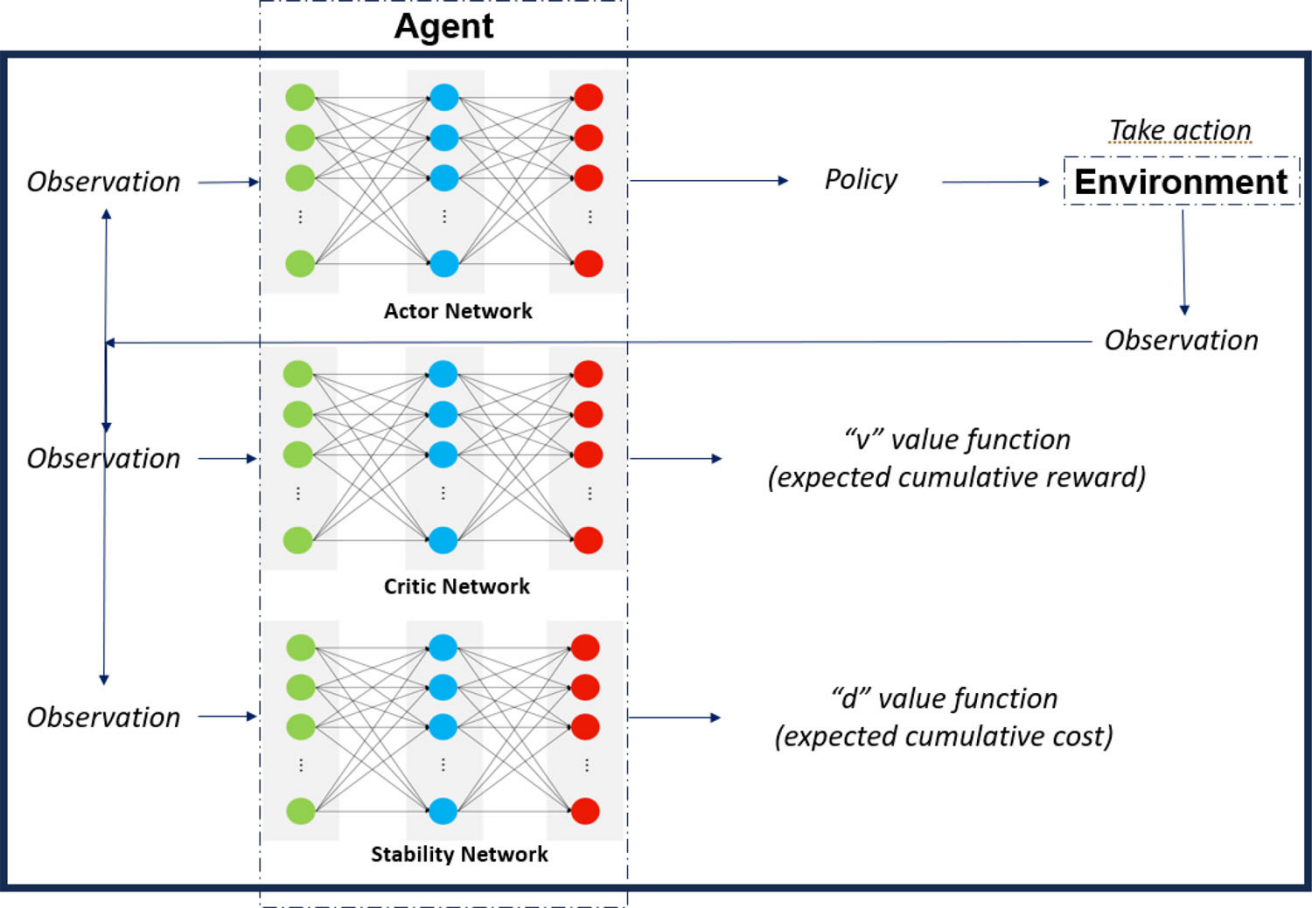
The AWAC framework

In the proposed framework, an extra stability network, $d_z(s)$, is introduced, parameterised by *z*, which approximates the expected cumulative cost, as shown in Fig. 8. In this way, a disadvantage, $D(s_t,a_t)$, is calculated as presented in (4). The role of $D(s_t,a_t)$ is to guide the agent towards stability. However, it should not subsume the role of $A(s_t,a_t)$ that guides the agent towards good performance. Hence, the impact of $D(s_t,a_t)$ is weighted by a factor, $\omega$, with $0.1 \le \omega \le 0.5$, such as the new, total advantage, $A'(s_t,a_t)$, can be calculated in (5). As a result, the update steps for the neural networks can be calculated in (6), (7) and (8), with $\eta$ denoting the learning rate of the stability network.

4 $$\begin{aligned} D(s_t,a_t)= &  c_{t+1} + \gamma d_z(s_{t+1})-d_z(s_t) \end{aligned}$$

5 $$\begin{aligned} A'(s_t,a_t)= &  A(s_t,a_t) - \omega D(s_t,a_t) \end{aligned}$$

6 $$\begin{aligned} \Delta \theta= &  \alpha \nabla _\theta (ln\pi _\theta (s_t,a_t))A'(s_t,a_t) \end{aligned}$$

7 $$\begin{aligned} \Delta w= &  \beta \nabla _w(v_w(s_t))A(s_t,a_t) \end{aligned}$$

8 $$\begin{aligned} \Delta z= &  \eta \nabla _z(d_z(s_t))D(s_t,a_t) \end{aligned}$$

Specifically, $\omega$ is defined as a sigmoid function, $\sigma (\xi )$, where $\xi$ is the intermediate variable between the agent’s training progress and the $\omega$ value allocation. The definition of the designed sigmoid function is presented in (9).

In order to exploit the meaningful region of the sigmoid function and maintain the effectiveness in the $\omega$ adjustment, the aim is to stay outside the saturation areas of the sigmoid function. Therefore, a limit is applied to $\xi$, such as $-5\le \xi \le 5$. The training process begins by allocating an initial value to the intermediate variable, $\xi =-5$, which leads to $\omega = 0.1$. When $\omega$ is close to 0.1 (initial value), rapid $\omega$ changes are avoided, since the agent is still exploring and it is allowed to interact more freely with the environment. At the same time, when $\omega$ is close to 0.5, rapid $\omega$ changes are also avoided, since the agent should persistently be discouraged to repeat poor policies. In other cases, the behaviour of the $\omega$ value changes is close to linear.

9 $$\begin{aligned} \omega =\sigma (\xi )=0.1 + \frac{0.4}{1 + e^{-\xi }} \end{aligned}$$

During training, the current mean expected cost, $EC_{curr}$, is estimated and compared against the mean expected cost of the previous training update, $EC_{prev}$, in order to monitor the direction of the training’s stability. Then, an increment, $\Delta \xi _{incr}$, is applied if the cost is heading towards higher values, and a decrement, $\Delta \xi _{decr}$, otherwise, in the way presented in (10) and (11) respectively. This tuning of the $\xi$ variable is always within the limitation of $-5\le \xi \le 5$. The pseudo-code of the proposed algorithm is shown in Algorithm 1.

10 $$\begin{aligned} \Delta \xi _{incr}= + \frac{1}{2}\frac{EC_{curr}}{EC_{prev}} \end{aligned}$$

11 $$\begin{aligned} \Delta \xi _{decr}= - \frac{1}{2}\frac{EC_{prev}}{EC_{curr}} \end{aligned}$$

**Algorithm 1:**
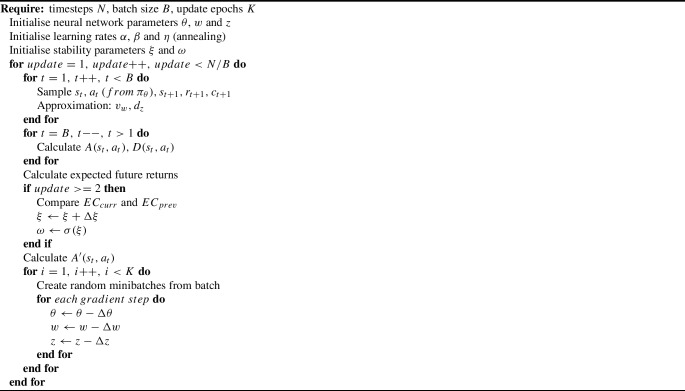
Adaptive weighted actor critic

**Table 2:** RL implementation summary

Case study	Action space Box(1,)	State space Box(3,)	Episode duration
Fixed target	Power [250, 300]	Temperature, power, height	35 timesteps (layers)
Tracking target	Power [250, 350]	Temperature, power, height	35 timesteps (layers)

Intuitively, instead of using the AWAC method, one could attempt to manipulate the reward function accordingly, i.e. $r'_{t+1} = r_{t+1} + \omega \,c_{t+1}$. The reward function manipulation is a straightforward approach to provide a purely heuristic stability improvement, however, the proposed AWAC method has some significant benefits, which are discussed below:

- In AWAC, the $\omega$ factor is auto-tuned during training and it is constantly updated according to the training’s progress. With reward manipulation, this tuning would have to be manually implemented, based on a series of necessary experiments beforehand that would determine an arguably sub-optimal value for $\omega$.
- In AWAC, the training of the critic network and the training of the stability network occur independently. The stability network only influences the policy update without complicating further the critic network’s training. With reward manipulation, however, both the performance metric and the stability metric would be included in the reward function, hence they would be both included in the training of the critic’s network.
- In AWAC, the reward graph is straightforward to interpret since the reward consists solely of the performance metric of the problem. With reward manipulation, the reward graph would become more complex to understand, hence it would be challenging to investigate the contribution of each reward factor in the agent’s performance.

A substantial benefit of AWAC is that its RL framework does not require any additional hyperparameters to be manually tuned. Thus, it is simple to implement and interpret its performance. The only intervention by the user is the construction of a stability cost function, which guides the agent towards its goal, assisting the originally constructed reward function within the RL framework. The construction of a stability cost function requires similar intuition to the one corresponding to the construction of any reward function, hence, there is no inherently added complexity in the proposed method. One could include this stability cost function in the originally constructed reward function of SAC and gain similar results with AWAC. However, this would raise a number of interpretation issues and tuning challenges. Hence, the benefit of AWAC is that it yields a more intuitive approach to introduce stability in the RL training than reward manipulation.

## SLM process control results

### Control problem

In this section, the benefits and the challenges of layer-wise control in SLM are demonstrated, via case studies of applications in symmetric parts. The control is implemented by varying the power, layer-by-layer, in order to achieve the desired average layer temperature of the meltpool. The geometry of the part plays crucial role regarding the level of challenge in this control task. If the layers of the part are long and wide enough, then the heat dissipates effectively and the heat accumulation observed among layers is small. However, when addressing thinwall structures (e.g. thin design features in heat exchangers), the heat accumulation observed among layers is significant, and changing the power according to a control law can be crucial for the quality of the produced part. Hence, in this demonstration the focus is on a thinwall geometry.

Specifically, the geometry of the part is a cuboid, with a base that consists of 4 tracks, with 10 mm length each, resulting in a thinwall geometry. The cuboid consists of 35 layers. The simulation model is set up to take a value of power for each layer as an input, and produce for this layer a time series of thermal history, consisting of 800 points. This time series per layer is then averaged to be used as a performance indicator (and target for control). Each layer comprises a timestep from the controllers’ perspective. When applying RL, in terms of RL notation and terminology, see Sutton and Barto (1998), the whole build is referred to as an episode. Hence, it follows that the whole build is an episode consisting of 35 timesteps (35 layers). The RL implementation details for the following case studies are presented in Table 2.

Three different control approaches are compared; the state of the art SAC reinforcement learning, the stable approach AWAC reinforcement learning, and these are benchmarked against a PID controller. Regarding the RL techniques, SAC and AWAC, the agents undergo a training process before they come up with their resulting policy for the control problem. Hence, it is essential to compare the SAC and AWAC agents’ training process and assess the impact of AWAC in the stability of the training. The improvement in stability is measured with regards to reduction in RL training variance, both overall and during pre-convergence training periods, while achieving higher or at least the same reward values. After the training is completed, the resulting policies of SAC and AWAC are compared, against the control policy of a carefully tuned PID controller. The training of SAC and AWAC is a stochastic process, hence the resulting policy of the agents is different for each time the same experiment is run. For the PID, however, the resulting policy is always the same (deterministic) for the same experiment. Hence, in order to compare the SAC and AWAC training processes, multiple experiments are run and statistical analysis is performed. For comparison, the control policy for the PID is contrasted against the average resulting policy for the SAC and the AWAC agents (rather than the best resulting policy). The control policies are tested in two scenarios, in a target tracking setting, where the target is fixed, as well as a setting where the target varies with time. The hyperparameters used for the SAC and the AWAC agent are shown in Table 3.

**Table 3:** Hyperparameters for SAC and AWAC training

RL agents	Learning rates	Buffer/batch size	Discount factor	Entropy coefficient/$\omega$ factor
SAC	3e-4	1e6	0.99	auto
AWAC	3e-4	2048	0.99	auto

### Thinwall control with fixed control target

The first case study is process control for a thinwall geometry, with the aim of maintaining the average layer temperature constant throughout the build. $T_{melt}$ is defined as the average layer temperature observed and $A_{melt}$, as the average meltpool area observed during the simulated build of a layer. The desired temperature is set to be $T_{target}$= 2308 K and the SAC controller framework is designed as follows:

- Action space: The action space is a continuous space and it includes only the power of the laser beam, $250\,\text {W} \le P\le 300$ W.
- State space: The state space is a continuous space. It consists of the observed average layer temperature, $T_{melt}$, the power, *P*, that was used to achieve this temperature, and the part’s current height (layer).
- Reward function: The reward function plays a crucial role to RL training. Based on the work of Ogoke and Farimani (2021), the reward function r, per layer, is formulated as:
  12 $$\begin{aligned} r = 1 - \left| \frac{T_{target}-T_{melt}}{100}\right| \end{aligned}$$

In order to implement the AWAC control approach, the same action space, state space and reward function are used as in the case of SAC. All the environment’s definitions and assumptions remain the same. However, there is a need for a stability metric to indicate if the agent is far or close to the control target. The stability metric in this case study is chosen to be the meltpool area, since the meltpool area is correlated with the meltpool temperature, which is the control target variable (one may select a different stability metric). The desired temperature is still set to be $T_{target}$= 2308 K and the corresponding desired average meltpool area is calculated to be $A_{target}$= 5.44$\text {e}{-8}\hbox {m}^{2}$. For the cost function c, per layer, a quadratic Lyapunov term is constructed, as it is also common in control theory, see Sistu and Bequette (1996). In this case, it is formulated as:

13 $$\begin{aligned} c = \left( \frac{A_{target}-A_{melt}}{0.01\text {e}{-8}}\right) ^2 \end{aligned}$$

**Fig. 9:**
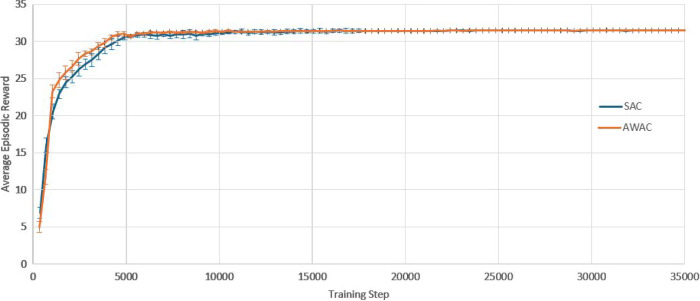
Training curves of the SAC and the AWAC agents. Fixed target for the average layer temperature

10 separate experiments are run for each agent (SAC and AWAC). Figure 9 depicts the training process of the SAC and AWAC agent. The average reward and the standard deviation are shown by the dense line and the error bars respectively. It is observed that the agents achieve high levels of reward, since they reach a maximum higher than 31.5 out of 35 (maximum theoretically possible), hence higher than 90% training performance. The AWAC agent seems to outperform the SAC agent in stability and overall robustness of the training. More specifically, the comparison metrics and the results for the training are shown in Table 4. Similar to the step response settling time in control theory, the settling time here refers to the timestep in which the agent reaches 95% of the maximum reward and stays consistently above the 95% for the rest of the training (convergence). The mean settling time std refers to the mean of the standard deviations calculated from the beginning of the training until the settling time.

**Table 4:** Fixed target training comparison between SAC and AWAC

RL agents	Mean reward	Mean of std	Max reward	Settling time	Mean settling time std
SAC	30.37	0.24	31.54	4550	0.80
AWAC	30.55	0.15	31.54	4200	0.70

All values correspond to the average of 10 runs

**Fig. 10:**
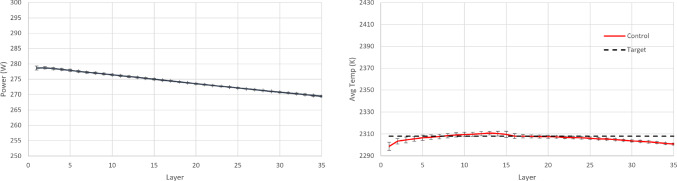
Resulting policy of the SAC agent with fixed target. Training stopped at 33.3% convergence timesteps

**Fig. 11:**
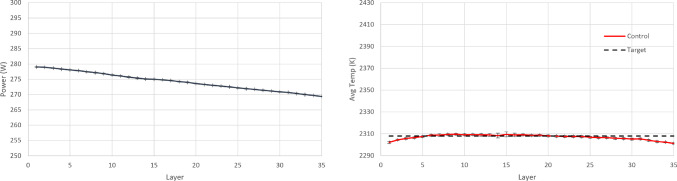
Resulting policy of the AWAC agent with fixed target. Training stopped at 33.3% convergence timesteps

**Fig. 12:**
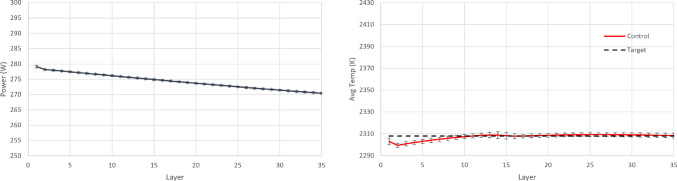
Resulting policy of the SAC agent with fixed target. Training stopped at 66.6% convergence timesteps

**Fig. 13:**
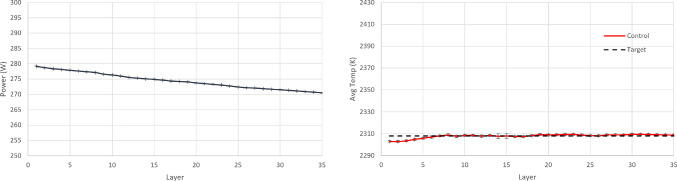
Resulting policy of the AWAC agent with fixed target. Training stopped at 66.6% convergence timesteps

At this point, it is worth investigating the training process of SAC and AWAC more in depth, in order to gain intuition and fully understand the benefits of the AWAC approach. As observed in Table 4, the maximum rewards achieved in the two training processes show no significant difference between SAC and AWAC, while both training processes converge to the same reward values. Hence, it is expected that the resulting policies of the two approaches after the training completion show no significant difference. However, after the early, preliminary training steps (where interactions are mostly random for both agents), it is observed that AWAC reaches consistently higher rewards than SAC during the pre-convergence period. In a realistic scenario, where computational cost and resources availability could be critical factors, one might not be able to train the RL agents until full convergence. Hence, it is plausible that the training process would have to stop during the pre-convergence period. For this reason, an additional experiment is run, where in the 10 separate training processes conducted before for each agent, see Fig. 9, the training is terminated before the settling time, and the resulting policy of the two agents is investigated. Specifically, the training is stopped at 33.3% convergence and at 66.6% convergence and the resulting policies and temperatures of SAC and AWAC are shown in Figs. 10, 11, 12 and 13. In the 33.3% scenario, it is observed that the AWAC agent comes up with a better policy to maintain the average layer temperature at the desired value, reaching a mean of 2306.9 K, with a mean absolute error of 1.87 K. The SAC agent also reaches satisfactory temperatures, with a mean of 2306.2 K, however, the mean absolute error is 2.57 K, which is approximately 38% worse than AWAC. In the 66.6% scenario, it is observed once more that the AWAC agent comes up with a better policy, reaching a mean of 2307.9 K, with a mean absolute error of 1.22 K. The SAC agent also reaches satisfactory temperatures, with a mean of 2307.2 K, however, the mean absolute error is 1.74 K, which is approximately 43% worse than AWAC. In conclusion, the fact that AWAC reaches consistently higher rewards than SAC, in a more stable manner, has significant impact in the resulting policy of the two agents, and this impact is particularly shown in cases where there are limitations in computational resources and cost.

**Fig. 14:**
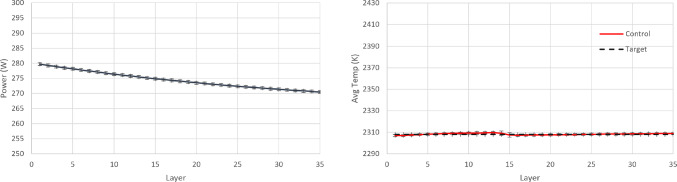
Resulting policy of the SAC agent with fixed target

**Fig. 15:**
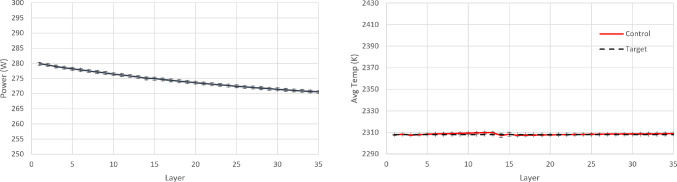
Resulting policy of the AWAC agent with fixed target

**Fig. 16:**
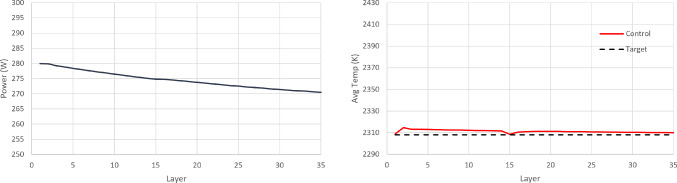
Resulting policy of the PID with fixed target

In order to implement PID control, parametric optimisation via gradient descent is used for tuning the P, I, and D terms. These terms are then further fine-tuned heuristically which results in a further performance increase. The final values of the control law terms are $K_P=5.58\text {e}{-}10$, $K_I=1.01\text {e}{-}13$ and $K_D=-1.9\text {e}{+}01$. The resulting policy (from RL completed training and PID tuning) and the achieved temperature in each layer are shown in Figs. 14, 15 and 16. It is argued that both RL controllers manage to maintain the average layer temperature in the desired value in a satisfactory manner throughout the build, outperforming the PID controller. It is noticed that the PID controller follows a reasonable policy of gradually decreasing power. However, it does not reach the desired value as effectively and consistently as the RL controllers, demonstrating a slight offset from the desired value. The offset challenge is a well-known challenge in PID controllers, see Shaw (2003). The full list of comparison metrics and the results for the controllers are shown in Table 5.

### Thinwall control with tracking control target

The second case study attempts to challenge the controller, in the sense that the control target is no longer fixed. This could be the case, for example, when one wishes to create bespoke material microstructure and local properties via manipulating the cooling rate between layers. The desired temperature, $T_{target}$, now varies with the part’s height, as seen in the results in Fig. 18. Hence, the way the RL frameworks are defined is altered, as follows:

- Action space: The action space is a continuous space and it includes only the power of the laser beam, 250 W $\le P\le$ 350 W.
- State space: The state space is a continuous space. It consists of the observed average layer temperature, $T_{melt}$, the power, *P*, that was used to achieve this temperature, and the part’s current height (layer).
- Reward function: The reward function plays a crucial role to RL training. Based on the work of Ogoke and Farimani (2021), the reward function r, per layer, is formulated as:
  14 $$\begin{aligned} r = 1 - \left| \frac{T_{target}-T_{melt}}{100}\right| \end{aligned}$$

As the control target is no longer fixed, the stability metric target for AWAC needs to be redefined accordingly. Specifically, a step change to the meltpool area target is introduced that corresponds to each new meltpool temperature target, as $A_{target}=5.44\text {e}{-8}\hbox {m}^{2}$, $A_{target}=6.06\text {e}{-8}\,\hbox {m}^{2}$, $A_{target}=5.10 \text {e}{-8}\,\hbox {m}^{2}$, $A_{target}=5.91 \text {e}{-8}\,\hbox {m}^{2}$, $A_{target}=6.40 \text {e}{-8}\,\hbox {m}^{2}$, $A_{target}=5.26\text {e}{-8}\,\hbox {m}^{2}$, and $A_{target}=5.44 \text {e}{-8}\,\hbox {m}^{2}$. For the cost function c, per layer, a quadratic Lyapunov term is constructed, as it is also common in control theory, see Sistu and Bequette (1996). In this case, it is formulated as:

15 $$\begin{aligned} c = \left( \frac{A_{target}-A_{melt}}{0.01\text {e}{-8}}\right) ^2 \end{aligned}$$

**Fig. 17:**
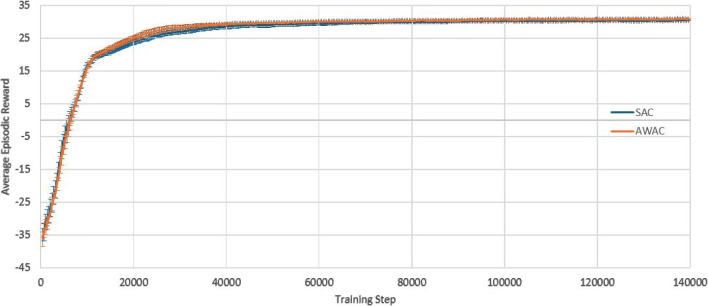
Training curves of the SAC and the AWAC agents. Tracking target for the average layer temperature

10 separate experiments are run for each agent (SAC and AWAC). Figure 17 depicts the training process of the SAC and AWAC agent. The average reward and the standard deviation are shown by the dense line and the error bars respectively. It is observed that the agents achieve high levels of reward, since they reach a maximum higher than 30.6 out of 35 (maximum theoretically possible), hence higher than 87% training performance. The AWAC agent seems to outperform the SAC agent in stability and overall robustness of the training. More specifically, the comparison metrics and the results for the training are shown in Table 6. Similar to the step response settling time in control theory, the settling time here refers to the timestep in which the agent reaches 95% of the maximum reward and stays consistently above the 95% for the rest of the training (convergence). The mean settling time std refers to the mean of the standard deviations calculated from the beginning of the training until the settling time.

**Table 5:** Fixed target results comparison of PID, SAC and AWAC

Controller	Mean temperature (K)	Mean temperature error (K)
SAC	2308.27	0.65
AWAC	2308.39	0.62
PID	2311.24	3.24

SAC and AWAC values correspond to the average of 10 runs

**Table 6:** Tracking target training comparison between SAC and AWAC

RL agents	Mean reward	Mean of std	Max reward	Settling time	Mean settling time std
SAC	26.28	0.54	30.65	44,800	0.98
AWAC	26.51	0.35	30.83	40,950	0.94

All values correspond to the average of 10 runs

**Fig. 18:**
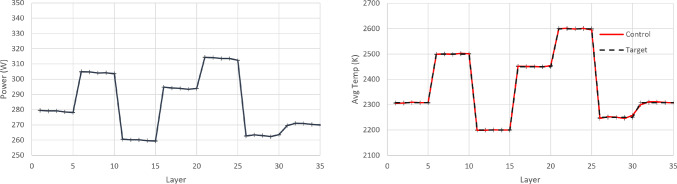
Resulting policy of the SAC agent with tracking target

**Fig. 19:**
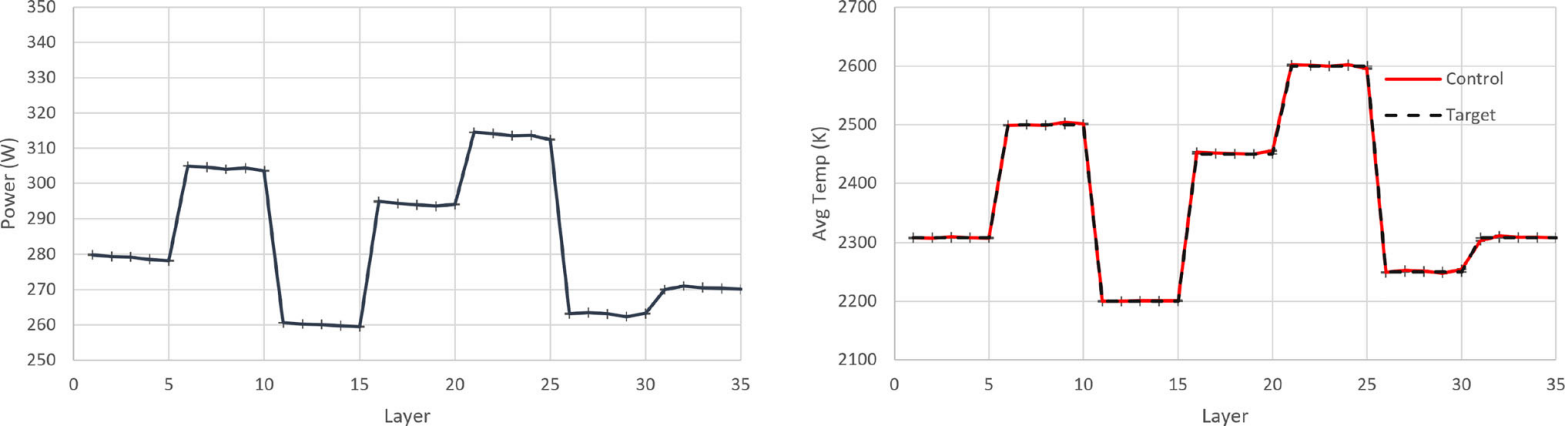
Resulting policy of the AWAC agent with tracking target

**Fig. 20:**
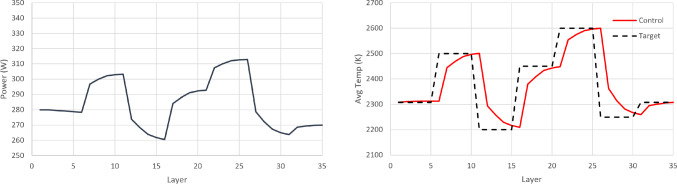
Resulting policy of the PID with tracking target

For the PID controller, the same formulation and tuning are used as in the earlier case study (fixed control target). The resulting policy and the achieved temperature in each layer are shown in Figs. 18, 19 and 20. It is argued that both RL controllers manage to maintain the average layer temperature in the desired value in a satisfactory manner throughout the build, outperforming the PID controller. The PID controller demonstrates a delay in the action it takes when there is a change in the control target. This delay is expected (and a known drawback in PID control methods), see Shaw (2003), since the controller does not know about the change in target, until this is fed back indirectly via the error signal. In contrast, the RL controllers demonstrate no delay and they follow the target effectively. More specifically, the comparison metrics and the results for the controllers are shown in Table 7.

## Discussion and future research directions

For the first time in the literature, in this study we demonstrate the benefits of a RL process control framework for multiple layers (complete 3D PBF parts) and we highlight the importance of stability during training. The presented case studies confirm the effectiveness of the proposed control framework, directly addressing heat accumulation issues while demonstrating effective overall process control. Based on simple 3D part geometries, SAC and AWAC outperform a tuned PID controller. Moreover, when comparing the RL algorithms’ training, we confirm the benefits of the proposed AWAC approach regarding stability and consistent performance, which can be key factors for satisfactory manufacturing results and practical applications.

Despite the satisfactory control performance of RL in our case studies, there are still important challenges that prevent RL from entering the control mainstream in the metal AM industry. As our case studies show, the RL agent needs to be trained before it derives a control policy. However, there is no established way to predict if the training is going to be successful. Further research, new methods and new analyses are needed in the areas of convergence guarantees, constraint satisfaction, and control performance guarantees, to create more trustworthy implementations of RL (which are required for critical applications).

From a process perspective (PBF), a noteworthy challenge is the development of advanced control strategies, for example to create bespoke microstructure and localised part properties. While this work focuses on a layer-by-layer control approach, there is a need to explore track-by-track and point-by-point control frameworks. However, these would require more complex models, that would have higher computational demands. We also expect that the identification of good control policies would be more challenging, hence this would require more effective and efficient RL methods.

**Table 7:** Tracking target results comparison of PID, SAC and AWAC

Controller	Action delay	Mean temperature error (K)
SAC	No	1.95
AWAC	No	1.63
PID	Yes	59.05

SAC and AWAC values correspond to the average of 10 runs

Finally, in this work, we formulate the meltpool behaviour in a deterministic fashion. However, in a real-world PBF process, the meltpool behaviour is not deterministic, since there is uncertainty within the manufacturing process and sensitivity limitations in the monitoring capability and systems. In order to make the PBF simulation more realistic and test the controllers’ capabilities, there is a need for testing against monitoring noise and other process disturbances. Hence, further research is required towards validation of simulation results with real-world experiments, as well as practical implementation for a range of part geometries and materials. This would also lead to further research work in RL, towards methods that are more tolerant to uncertainty and disturbances.
